# Data on the effects of The-Optimal-Lymph-Flow program on lymphedema symptoms in breast cancer survivors

**DOI:** 10.1016/j.dib.2023.109278

**Published:** 2023-05-28

**Authors:** Mei Rosemary Fu, Xinwen Du, Yuan Li, Lan Fu, Huaying Chen, Xiaoxia Zhang, Yuping Shui, Aihua Zhang, Xianqiong Feng

**Affiliations:** a School of Nursing, The George Washington University, Washington, D.C. 20006, USA; b Department of Hematology, West China Hospital, Sichuan University/West China School of Nursing, Sichuan University, Chengdu 610041, China; c Department of Neonatology Nursing, West China Second University Hospital, Sichuan University/West China School of Nursing, Sichuan University, Chengdu 610041, China; d Cancer Center, West China Hospital, Sichuan University/West China School of Nursing, Sichuan University, Chengdu 610041, China; e Head & Neck Oncology Ward, Cancer Center, West China Hospital, Sichuan University/West China School of Nursing, Sichuan University, Chengdu 610041, China; f Department of Operating Room, West China Hospital, Sichuan University/West China School of Nursing, Sichuan University, Chengdu 610041, China; g West China School of Nursing, Sichuan University, Chengdu 610041, China

**Keywords:** Breast cancer, Lymphedema, Lymphatic exercises, Symptom management, Mobile health, Randomized clinical trial

## Abstract

A substantial proportion of more than 50% of breast cancer survivors, who remain undiagnosed with lymphedema, encounter a daily struggle with the presence of multiple and concomitant lymphedema associated symptoms (i.e., lymphedema symptoms). The-Optimal-Lymph-Flow (TOLF) program was developed based on physiological-cognitive-behavioral principles to educate breast cancer survivors on effective self-care strategies. Physiologically, TOLF program was designed to stimulate lymphatic system to enhance lymph flow, thereby alleviating lymphedema symptoms and mitigating the risk and severity of lymphedema. The dataset presented in this article was obtained from a randomized clinical trial (RCT) that assessed the preventive effects of the TOLF program in improving lymphedema symptom experience and optimizing lymph fluid status among breast cancer survivors who were at higher risk for lymphedema.

Between January 2019 and June 2020, a RCT was conducted to recruit 92 eligible participants who were assigned randomly to either the TOLF group (intervention) or the arm mobility group (control). Demographic and clinical data were collected at baseline and updated over the study period. Outcome data were collected at baseline and three months after intervention. Study outcomes included lymphedema symptom experience (i.e., number, severity, distress of lymphedema symptoms, and impact on daily activities) and lymph fluid status. The Breast Cancer and Lymphedema Symptom Experience Index (BCLE-SEI) was utilized to assess lymphedema symptoms and circumferential arm measurement was utilized to estimate limb volume differences (a surrogate for lymph fluid status). The dataset based on the RCT allowed confirmation of positive effects of the TOLF intervention during early postoperative period. The dataset can be further utilized as a benchmark reference in clinical settings or experimental research to determine the effects of optimal lymphatic exercise dosage on lymphedema risk reduction and symptom alleviation as well as provide a basis for future research related to this topic.

**Specifications Table**

**Table:** 

Subject	Health and medical sciences
Specific subject area	Breast-cancer related lymphedema symptoms
Type of data	Table
How the data were acquired	During the first face-to-face research visit, patients' demographic and clinical data were collected by self-report and checked for completeness and accuracy through reviewing medical records. Outcome data on lymphedema symptoms and lymph fluid status using anthropometric measurements were obtained at both study baseline and 3 months post-intervention. The questionnaires and corresponding code instructions can be found in the Mendeley repository.
Data format	Raw
Description of data collection	A total of 92 eligible patients completed the baseline demographic and clinical questionnaires. The BCLE-SEI was utilized to measure lymphedema symptoms. A TOLF mHealth system-based evaluation module was established, whereby participants were notified about the designated timing for each assessment, allowing them to gain access to the online questionnaires and complete them. Circumferential arm measurement was completed in-person to estimate limb volume differences.
Data source location	Institution: West China Hospital of Sichuan University City/Town/Region: Chengdu Country: China
Data accessibility	Repository name: Mendeley Data Data identification number: 10.17632/zhyb98cz6w.1 Direct URL to data: https://data.mendeley.com/datasets/zhyb98cz6w
Related research article	Du X, Li Y, Fu L, Chen H, Zhang X, Shui Y, Zhang A, Feng X, Fu MR. Strategies in activating lymphatic system to promote lymph flow on lymphedema symptoms in breast cancer survivors: A randomized controlled trial. *Front Oncol*. 12 (2022) 1015387. https://doi.org/10.3389/fonc.2022.1015387

## Value of the Data


 

• The data based on a RCT provides the reference for lymphedema symptom experience and limb volume differences in breast cancer survivors who are at higher risk of lymphedema.
• The data offers healthcare providers and researchers with references for clinical implementation or experimental studies to determine the optimal dose and effects for breast cancer survivors to reduce symptoms and lymphedema risk.
• The information obtained from the data will help enhance awareness of the distress caused by lymphedema symptoms among breast cancer survivors in the early postoperative period.
• The data can provide a basis for further studies on self-care strategies for alleviation of lymphedema symptoms and optimization of lymph fluid levels.

## 1 Objective

Globally, breast cancer is the most prevalent malignancy in women [1]. Lymphedema is a debilitating complication from breast cancer treatment, impacting at least one-fourth of the breast cancer survivors and causing multiple distressing symptoms [2,3]. The-Optimal-Lymph-Flow (TOLF) program is a web- and mobile-based digital system designed to empower patients with self-care strategies to promote lymph flow for effective management of lymphedema symptoms and risk reduction of lymphedema. This dataset was generated from a RCT [4] that aimed to determine the preventive effects of the TOLF program on improving experience of lymphedema symptom and optimizing lymph fluid status among breast cancer survivors who had higher risk of lymphedema.

## 2 Data Description

Patients were recruited from West China Hospital of Sichuan University, an accredited, nationally ranked academic medical center. Ninety-two breast cancer survivors were recruited and 88 participants successfully completed the three-month TOLF intervention. The participant flow chart was published and outlines the flow of participants through the study [4].

The raw data have been uploaded in *.xlsx format and organized into five sheets within an Excel spreadsheet. Table 1 gives an overview of the dataset composition.

**Table 1:** Overview of the dataset composition.

**Sheet 1. Sample characteristics (at baseline)**		

Column A: Participant ID	Column B: Group assignment	Column C: Age
Column D: Level of education	Column E: Marital status	Column F: Employment status
Column G: Living status	Column H: Dominant hand	Column I: Perceived household incomes
Column J: Time since diagnosis	Column K: Affected arm	Column L: Types of surgery
Column M: Axillary lymph node dissection	Column N: Sentinel lymph nodes biopsy alone	Column O: Chemotherapy
Column P: Radiotherapy		

**Sheet 2 & Sheet 3. Symptom experience (at baseline & after the 3-month intervention)**		

Column A: Participant ID	Column B: Group assignment	Column C: Number of symptoms
Column D: Number of symptoms	Column E: Number of symptoms	Column F: the impact of lymphedema symptoms on patients’ daily living functions

**Sheet 4 & Sheet 5. Limb volume (at baseline & after the 3-month intervention)**		

Column A: Participant ID	Column B: Group assignment	Column C∼R: Left upper limb volume measured at 4 cm intervals from the wrist to the shoulder
Column S∼AH: Right upper limb volume measured at 4 cm intervals from the wrist to the shoulder	Column AI: Limb volume difference (Is the interlimb volume difference ≥ 5% at any of the measurement locations?)	

## 3 Experimental Design, Materials and Methods

This dataset was generated from a RCT with a Clinical Trial Registration identifier of ChiTR1800016713 [4]. Potential participants underwent screening for lymphedema risk at one month after surgical treatment. Those patients who had not been diagnosed with lymphedema but reported at least five lymphedema symptoms were classified as having a higher risk of lymphedema [5]. Those patients who were classified as having a higher risk were invited to participate in the RCT. Higher risk patients aged 18 to 80 years were considered eligible if they (a) were nonpregnant; (b) received breast cancer surgical treatment for the first time; (c) had access to an internet-enabled device (e.g., smartphone, tablet, or computer); and (d) willing to participate and sign the written consent to the study. Patients were excluded if they had (a) delayed healing of incisions; (b) local or distant metastases; (c) a history of surgery or trauma affecting the affected axilla or arm; (d) severe mental illness or cognitive impairment; (e) severe cardiac or renal insufficiency; or (6) lymphedema not related to breast cancer.

Participants of the study were allocated randomly to either the TOLF intervention group or the arm mobility control group. Participants in the intervention group had full access to the TOLF platform to learn all relevant content. The core component of the TOLF intervention is the therapeutic lymphatic exercises [4,6]. Patients were instructed to learn and follow the 8-minute TOLF lymphatic exercises via avatar video simulations with step-by-step instructions. During the study period, patients in the intervention group were required to perform the lymphatic and limb mobility exercises at least two times per day to stimulate lymphatic fluid flow and facilitate shoulder and arm mobility. Meanwhile, control group's patients had no access to the core TOLF lymphatic exercises; they were only offered the access to the limb mobility exercises. The participants, outcome assessors, and statistician were kept blinded to the treatment allocation throughout the trial.

The demographic and clinical characteristics of the participants were collected through self-report and checked for completeness by reviewing medical records. Outcome data were collected at baseline and study endpoint and anthropometric measurements were obtained in-person. A TOLF mHealth system-based evaluation module was established to collect self-reported data, whereby participants were notified about the designated timing for each assessment and instructed to complete online questionnaires at each assessment time.

The primary outcome was the experience of lymphedema symptoms which was operationalized as the number, severity, and distress of lymphedema symptoms, and assessed by self-report using the BCLE-SEI [7]. The BCLE-SEI is a two-part psychometric instrument. The first part assesses the occurrence of 24 lymphedema symptoms. Each symptom is scored on a 5-point Likert scale ranging from 0 (symptom not present) to 4 (very severe symptom) for severity, and could also be treated as a binary variable with "0" indicating the “symptom not present” and other options indicating the “symptom present” [7]. The second part assesses the symptom distress which is defined as the negative impact and suffering induced by an individual's encounter lymphedema symptoms, spanning the domains of daily living functions (i.e., activities of daily living or ADLs), social impact, sleep disturbance, sexuality, psychological impact, and self-conception [7].

The secondary outcomes were the impact of lymphedema symptoms on patients’ ADLs measured by a subscale of the BCLE-SEI and limb volume differences measured by circumferential arm measurement [5]. To ensure accuracy, a validated arm circumference measurement protocol was utilized to measure the circumferences of both limbs, which involved continuous measurements at 4 cm intervals from the wrist to the shoulder [8]. Limb volume was computed via the following formula $V=D(C_{1}^{2}+C_{2}^{2}+C_{1}C_{2})/12\pi$, where *D* is the distance between *C*_1_ and *C*_2_, and *C*_1_ and *C*_2_ denote the circumferences of two neighboring measurement locations [8]. A limb volume difference of ≥ 5% was used as the minimum threshold for limb volume difference, as an interlimb volume difference of this magnitude typically leads to lymphedema symptoms and impairment in ADLs [6].

Collected data were summarized and tabulated in an Excel spreadsheet, with each participant assigned a study ID to ensure the anonymity of data collection, storage, and analysis.

## Ethics Statements

This randomized clinical study was prospectively registered at the Chinese Clinical Trial Registry (ChiCTR1800016713), and received ethical approval from the Biomedical Ethics Committee of the West China Hospital, Sichuan University (2019/24). Each participant was informed about the study details, including the aim, procedure, expected duration, risks involved, benefits, and rights of refusal/withdrawal. All participants provided written informed consent prior to their inclusion in the study.

## CRediT Author Statement

**Mei Rosemary Fu:** Conceptualization, Methodology, Writing – Original draft preparation, Reviewing & Editing; **Xinwen Du:** Conceptualization, Methodology, Investigation, Formal analysis, Writing – original draft preparation; **Yuan Li:** Methodology, Validation, Writing – original draft preparation, Writing – review & editing; **Lan Fu:** Investigation; **Huaying Chen:** Investigation; **Xiaoxia Zhang:** Investigation; **Yuping Shui:** Methodology, Investigation; **Aihua Zhang:** Investigation; **Xianqiong Feng:** Conceptualization, Methodology, Validation, Writing – review & editing. All the authors have approved the final version of submission.

## Declaration of Competing Interest

The authors declare that they have no known competing financial interests or personal relationships that could have appeared to influence the work reported in this paper.

## Data Availability

Data on the effects of The-Optimal-Lymph-Flow program on lymphedema symptoms in breast cancer survivors (Original data) (Mendeley Data).

## Appendix Supplementary materials

Image, application 1

Image, application 2

Image, application 3
