# Supplementary material for: Data on the effects of The-Optimal-Lymph-Flow program on lymphedema symptoms in breast cancer survivors
Source: Data Brief. 2023 May 28;48:109278. doi: 10.1016/j.dib.2023.109278 (PMC10294092; doi:10.1016/j.dib.2023.109278)
Supplement: Supplementary file 1 [file mmc1.docx]

**Questionnaire**

**Part I. Demographic and clinical characteristics**

**Age (years):** ____

**Time since diagnosis (months):** ____

**Level of education:**

A. Primary school or below B. Middle school C. High school

D. Associate degree E. Bachelor’s degree or above

**Marital status:** A. Single/divorced B. Married

**Employment status:** A. Unemployed B. Employed

**Living status:** A. Live alone B. Live with family

**Dominant hand:** A. Left B. Right

**Perceived household incomes:**

A. Do not have enough to make ends meet B. Have enough to make ends meet

C. Comfortable: have more than enough to make ends meet

**Affected arm:** A. Left B. Right

**Types of surgery:** A. Lumpectomy B. Mastectomy

**Axillary lymph node dissection:** A. Yes B. No

**Sentinel lymph nodes biopsy alone:** A. Yes B. No

**Chemotherapy:** A. Yes B. No

**Radiotherapy:** A. Yes B. No

**Part II. Breast Cancer and Lymphedema Symptom Experience Index**

The following questions are about your symptom experiences in your affected limb in the past 4 weeks. The word “affected” means the same body side on which you received breast surgery or radiation. The word “limb” means the affected arm and hand. Have you had ___?

|  | **None** | **A little** | **Somewhat** | **Quite a bit** | **Very Severe** |
| --- | --- | --- | --- | --- | --- |
| Arm or hand swelling |  |  |  |  |  |
| Breast swelling |  |  |  |  |  |
| Chest wall swelling |  |  |  |  |  |
| Firmness in the affected limb |  |  |  |  |  |
| Tightness in the affected limb |  |  |  |  |  |
| Heaviness in the affected limb |  |  |  |  |  |
| Toughness or thickness of skin in the affected limb |  |  |  |  |  |
| Stiffness in the affected limb |  |  |  |  |  |
| Tenderness |  |  |  |  |  |
| Hotness/increased temperature in the affected limb |  |  |  |  |  |
| Redness in the affected limb |  |  |  |  |  |
| Blistering in the affected limb |  |  |  |  |  |
| Pain/aching/soreness |  |  |  |  |  |
| Numbness in the affected limb |  |  |  |  |  |
| Burning sensation in the affected limb |  |  |  |  |  |
| Stabbing in the affected limb |  |  |  |  |  |
| Tingling (pins and needles) in the affected limb |  |  |  |  |  |
| Fatigue in the affected limb |  |  |  |  |  |
| Weakness in the affected limb |  |  |  |  |  |

Have you had limited movement of your affected…?

|  | **None** | **A little** | **Somewhat** | **Quite a bit** | **Very Severe** |
| --- | --- | --- | --- | --- | --- |
| Shoulder |  |  |  |  |  |
| Elbow |  |  |  |  |  |
| Wrist |  |  |  |  |  |
| Arm |  |  |  |  |  |
| Fingers |  |  |  |  |  |

How much do your symptoms negatively affect your ability to do the following daily activities?

|  | **Did not do it** | **None** | **A little** | | **Somewhat** | **Quite a bit** | **A lot** |
| --- | --- | --- | --- | --- | --- | --- | --- |
| Cooking |  |  | |  |  |  |  |
| Using a knife to cut food |  |  | |  |  |  |  |
| Writing or typing or using computer |  |  | |  |  |  |  |
| Cleaning house |  |  | |  |  |  |  |
| Vacuuming |  |  | |  |  |  |  |
| Doing laundry |  |  | |  |  |  |  |
| Bathing self |  |  | |  |  |  |  |
| Taking care of children [holding a baby, changing diapers, feeding a child] |  |  | |  |  |  |  |
| Carrying or lifting heavy objects |  |  | |  |  |  |  |
| Yard work or gardening |  |  | |  |  |  |  |
| Dressing self |  |  | |  |  |  |  |
| Driving |  |  | |  |  |  |  |
| Making bed |  |  | |  |  |  |  |

How much do your symptoms negatively affect your normal social activities with family, friends, neighbors, or groups?

1. None
2. A little
3. Somewhat
4. Quite a bit
5. A lot

How much do your symptoms negatively affect you regular leisure activities?

1. None
2. A little
3. Somewhat
4. Quite a bit
5. A lot

How much do your symptoms negatively affect your mood or cause psychological distress?

|  | **None** | **A little** | **Somewhat** | **Quite a bit** | **A lot** |
| --- | --- | --- | --- | --- | --- |
| Frustration |  |  |  |  |  |
| Sadness |  |  |  |  |  |
| Guilty/self-blame |  |  |  |  |  |
| Worried |  |  |  |  |  |
| Irritability |  |  |  |  |  |
| Fear |  |  |  |  |  |
| Anger |  |  |  |  |  |
| Lonely |  |  |  |  |  |
| Helpless |  |  |  |  |  |
| Hopeless |  |  |  |  |  |
| Anxiety |  |  |  |  |  |
| Depression |  |  |  |  |  |

How much do your symptoms negatively change your perception of yourself?

1. None
2. A little
3. Somewhat
4. Quite a bit
5. A lot

[For example, people might view themselves as “loss of pre-cancer being,” “a person with pain, or swelling, or lymphedema,” “loss of independence”, a person who needs others’ help,” “not attractive, or not sexy, “disabled or handicapped”]

How many times do you wake up at night because of your symptoms?

1. None
2. Once at night
3. 2 times at night
4. 3 times at night
5. 4 or more times at night

How much do your symptoms negatively affect your sex life (performance of sex act) with your spouse or partner)?

1. No sex life
2. None
3. A little
4. Somewhat
5. Quite a bit
6. A lot

How much do your symptoms negatively affect your intimate or emotional relationship with your spouse or partner?

1. No spouse or partner
2. None
3. A little
4. Somewhat
5. Quite a bit
6. A lot

How much do your symptoms negatively affect your work outside the home (occupation)?

1. No job
2. None
3. A little
4. Somewhat
5. Quite a bit
6. A lot
