# Supplementary material for: Data on the effects of The-Optimal-Lymph-Flow program on lymphedema symptoms in breast cancer survivors
Source: Data Brief. 2023 May 28;48:109278. doi: 10.1016/j.dib.2023.109278 (PMC10294092; doi:10.1016/j.dib.2023.109278)
Supplement: Supplementary file 2 [file mmc2.docx]

**Code book**

**Group:** TOLF group = “1”, Limb mobility group = “2”

**Part I. Demographic and clinical characteristics**

**Level of education:** Primary school or below = “1”, Middle school = “2”, High school = “3”, Associate degree = “4”, Bachelor’s degree or above = “5”

**Marital status:** Single/divorced = “1”, Married = “2”

**Employment status:** Unemployed = “1”, Employed = “2”

**Living status:** Live alone = “1”, Live with family = “2”

**Dominant hand:** Left = “1”, Right = “2”

**Perceived household incomes:** Do not have enough to make ends meet = “1”, Have enough to make ends meet = “2”, Comfortable: have more than enough to make ends meet = “3”

**Affected arm:** Left = “1”, Right = “2”

**Types of surgery:** Lumpectomy = “1”, Mastectomy = “2”

**Axillary lymph node dissection:** Yes = “1”, No = “0”

**Sentinel lymph nodes biopsy alone:** Yes = “1”, No = “0”

**Chemotherapy:** Yes = “1”, No = “0”

**Radiotherapy:** Yes = “1”, No = “0”

**Part II. Breast Cancer and Lymphedema Symptom Experience Index**

None = “0”

A little = “1”

Somewhat = “2”

Quite a bit = “3”

Very Severe/A lot = “4”

Did not do it/ No sex life/ No spouse or partner/ No job = “0”

Once at night = “1”

2 times at night = “2”

3 times at night = “3”

4 or more times at night = “4”
